# Early detection of brainstem herniation using electroencephalography monitoring – case report

**DOI:** 10.1186/s12883-020-01988-7

**Published:** 2020-11-07

**Authors:** Naresh Mullaguri, Jonathan M. Beary, Christopher R. Newey

**Affiliations:** 1 Neurocritical Care, Division of Neurology, Department of Medicine, Prisma Health Greenville Memorial Hospital, University of South Carolina School of Medicine, Greenville, SC USA; 2 Neurobehavioral Sciences, A.T. Still University, Kirksville, MO USA; 3 Epilepsy Center, Neurological Institute, Cleveland Clinic Foundation, Cleveland, OH USA; 4 Division of Neurocritical Care, Cerebrovascular Center, Neurological Institute, Cleveland Clinic Foundation, Cleveland, OH USA

**Keywords:** Electroencephalography, Brain injury, Cerebral blood flow, Cerebral herniation

## Abstract

**Background:**

Continuous electroencephalography (cEEG) is an important neuromonitoring tool in brain injured patients. It is commonly used for detection of seizure but can also be used to monitor changes in cerebral blood flow. One such event that can cause a change in cerebral blood flow is imminent, cerebral herniation. cEEG monitoring and quantitative electroencephalography (QEEG) can be used as neurotelemetry to detect cerebral herniation prior to onset of clinical signs.

**Case presentation:**

We discuss two cases highlighting the use of cEEG in cerebral herniation accompanied by clinical examination changes. The first case is a patient with multiorgan failure and intracerebral hemorrhage (ICH). Given his coagulopathy status, his ICH expanded. The second case is a patient with intraventricular hemorrhage and worsening obstructive hydrocephalus. In both cases, the cEEG showed increasing regional/lateralized slowing. The Quantitative electroencephalography (QEEG) showed a decrease in frequencies, worsening asymmetry, decreasing amplitude and increasing burst suppression ratio corresponding with the ongoing herniation. Clinically, these changes on cEEG preceded the bedside neurological changes by up to 1 h.

**Conclusions:**

The use of cEEG to monitor patients at high risk for herniation syndromes may identify changes earlier than bedside clinical exam. This earlier identification may allow for an earlier opportunity to intervene.

## Background

Electroencephalography (EEG) is a vital and versatile component of modern neurotelemetry. Modern computer technology advances permit complex quantitative EEG spectral analysis. Beyond its more common application in the detection of seizure activity, EEG also has practical utility in detecting cerebral ischemia in vasospasm as well as providing a non-invasive means of intracranial pressure monitoring and functional stroke prognostication. We present novel case evidence for the utilization of EEG in the detection of cerebral herniation prior to clinical examination changes with review of recent literature.

## Case presentation

### Case 1

**Fig. 1:**
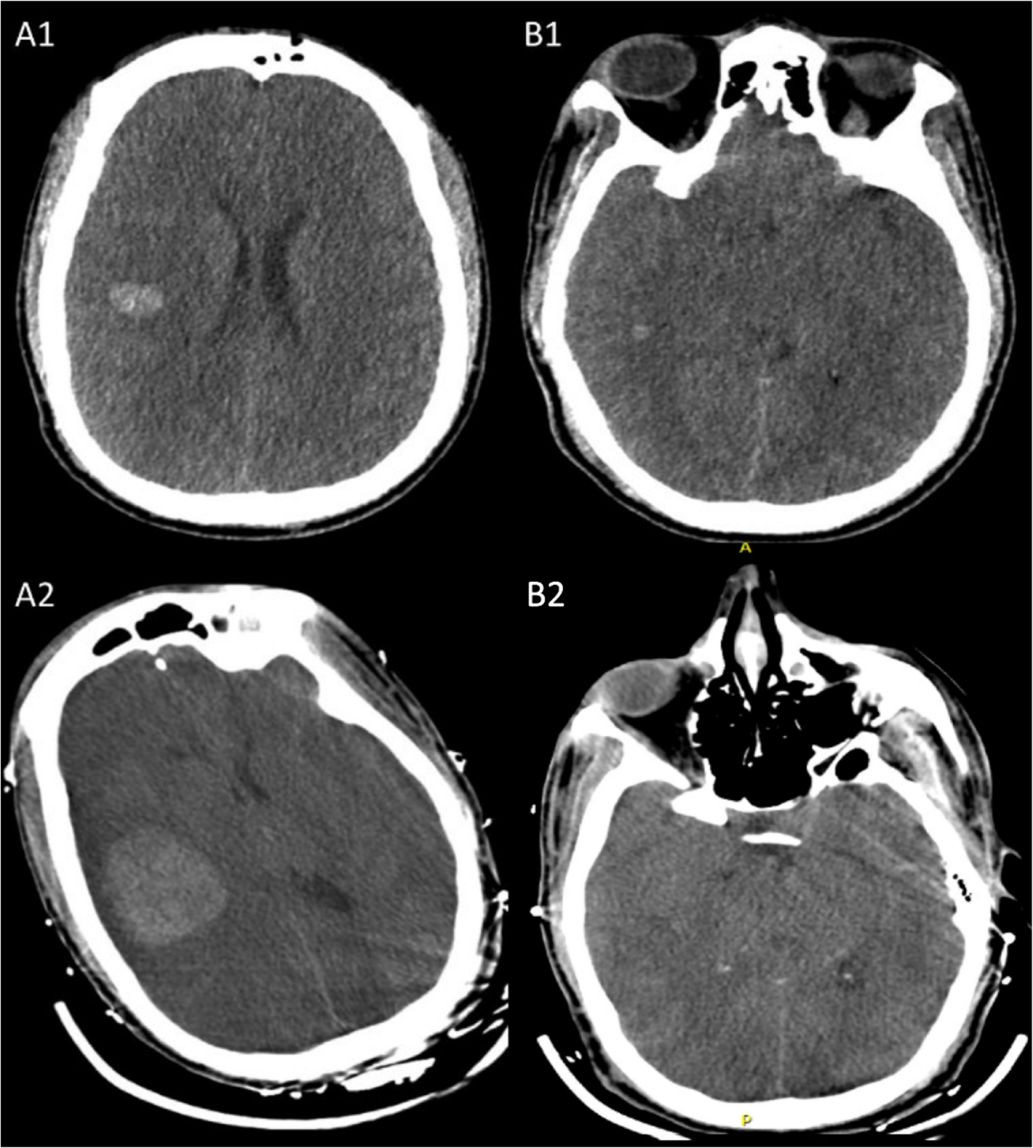
Computerized tomography of the brain – axial sections. A1, B1 – initial scan showing intracerebral hemorrhage in right frontal and temporal areas. The midbrain slice shows effacement of quadrigeminal cistern. A2, B2 – Day 3 scan showing large hemorrhage with intraventricular extension, severe cerebral edema with loss of grey-white differentiation, midbrain compression, and bilateral uncal herniation

A 46-year-old African American male presented to an outside hospital with 72 h of altered mental status. Past medical history was significant for chronic myelocytic leukemia in accelerated phase on dasatinib, ulcerative colitis, polysubstance abuse (cocaine, cannabinoids, and heroin), and splenic laceration status post splenectomy. On initial examination patient was combative and disoriented but was otherwise nonfocal. Initial blood work revealed leukocytosis (43,400 cells/mm3), INR > 5, creatinine 1.74 mg/dL, and lactic acidosis (pH 7.13, anion gap 30). CT brain showed multifocal intracerebral hemorrhages (ICH) in the right frontotemporal region (Fig. 1 (A1 and B1)). He was subsequently transferred to the neurocritical care unit with a coagulation profile suggestive of disseminated intravascular coagulation (fibrinogen – undetectable, d-dimer > 35,200 ng/mL, haptoglobulin < 10 mg/dL, and activated plasma thromboplastin time of 54.5 s. He was treated with cryoglobulin, fresh frozen plasma and platelet transfusions but developed tumor lysis syndrome (TLS) with elevated uric acid (12.2 mg/L, phosphorous 6.6 mg/dL. Repeat neuroimaging 6 h from initial scan showed hematoma expansion. The patient was started on intravenous hydration, allopurinol, hydroxyurea, rasburicase, and nilotinib. He developed acute respiratory failure and was intubated. Peripheral smear confirmed myelocytic leukemia with monocytic differentiation. Given the acute ICH, he was not a candidate for intensive chemotherapy regimen but pheresis for leukoreduction was initiated. He became hypotensive requiring multiple vasopressor medications and was started on broad spectrum antibiotics. Initial EEG showed continuous generalized slowing maximal in the right hemisphere suggestive of severe encephalopathy without seizure activity. Fibrinogen improved to 125 mg/dL. Repeat CT brain scan was stable.

**Fig. 2:**
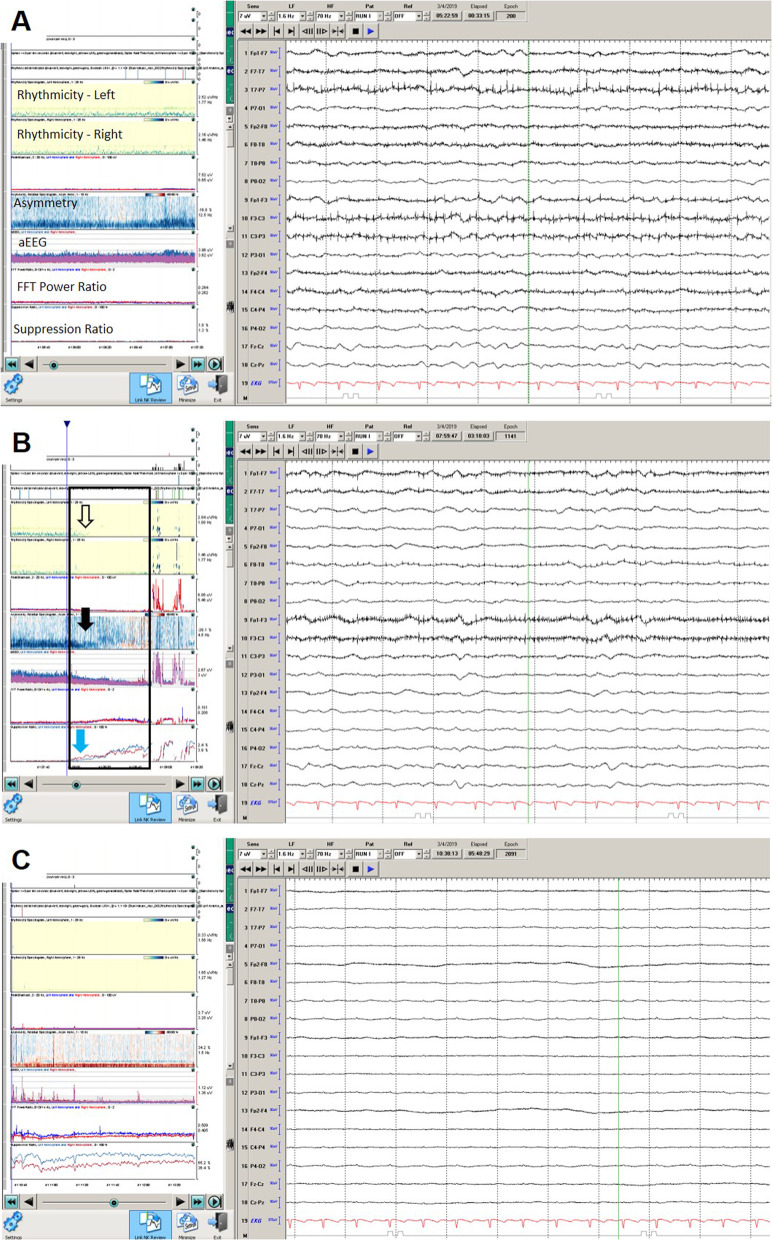
Continuous and quantitative electroencephalography changes of case 1. **a** Baseline EEG shows generalized slowing with a lateralized slowing in the left hemisphere generalized. **b** black box indicates the change in QEEG where rhythmicity in the right then left hemisphere drops out (open arrows) also noted, decrease in asymmetry of the left hemisphere (black arrow) and increasing in burst suppression ratio (blue arrow). **c** diffuse suppression

He was transferred to medical intensive care unit for management of multiorgan failure and TLS. On hospital day three at 8:00 am his right pupil became dilated and non-reactive. Repeat CT brain again was immediately obtained and showed stable right frontal hemorrhage although with multiple new bilateral supratentorial hemorrhages as well as uncal herniation and midbrain compression (Fig. 2 (A2 and B2)). Although at 09:30 am the left pupil also became dilated and non-reactive, neurosurgical intervention was deferred due to coagulopathy and overall poor prognosis. Approximately 1 h prior to left pupillary dilatation, His continuous electroencephalography (cEEG) showed worsening bilateral cortical dysfunction between 8:25–8:35 am (Fig. 2a). Quantitative Electroencephalography (QEEG) showed a transition from decrease in frequencies, changes in asymmetry, decrease in amplitude, and an increase in burst suppression ratio 2 h prior to onset of burst supression (Fig. 2b-c). No EEG reactivity was noted at this time. Despite hyperventilation and hyperosmolar therapy, cerebral herniation was not reversed. Due to poor prognosis, family requested comfort measures and the patient subsequently expired.

### Case 2

**Fig. 3:**
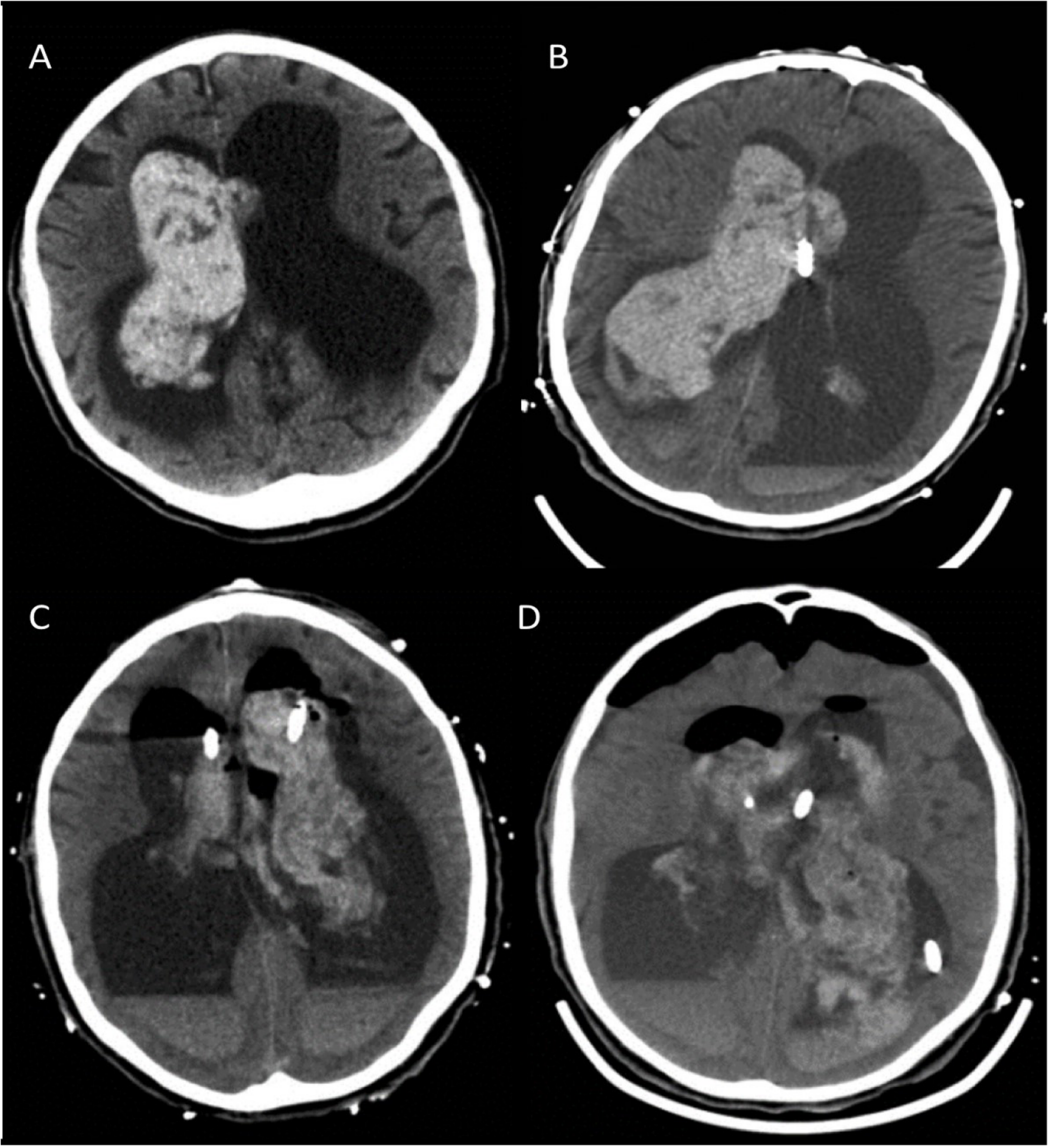
Computerized tomography of the brain axial section. **a** showing intraventricular hemorrhage (IVH) with hydrocephalus. **b** showing right frontal external ventricular drain (EVD) placement with no resolution of hydrocephalus. **c** showing post tPA scan with no resolution of IVH, interval placemnent of left frontal EVD. **d** showing post surgical changes of IVH evacuation, septostomy and new additional left parietal EVD placement with no radiological improvement of hydrocephalus

A 76-year-old Caucasian male presented from nursing home to outside hospital with chief complaint of confusion, loose stools, and chills. He had no focal neurological deficits. Past medical history was significant for spinal metastatic cancer of unknown primary origin, chronic communicating hydrocephalus with dementia, and baseline gait instability. CT brain showed isolated intraventricular hemorrhage (IVH) and hydrocephalus (Fig. 3a-d). He was transferred to the neurocritical care unit for further management. MRI and cerebral angiography imaging were noncontributory. Given worsening neurological exam, bilateral external ventricular drains (EVD) were placed. Repeated intraventricular dosing of recombinant tissue plasminogen activator resulted in minimal clinical improvement. The hospital course was complicated by electrographic seizures, paroxysmal sympathetic hyperactivity, syndrome of inappropriate anti diuretic hormone secretion, respiratory failure secondary to aspiration pneumonia, as well as EVD malfunction with spikes of intracranial hypertension. The neurological exam continued to be poor with intact brainstem reflexes and minimal spontaneous withdrawal in the upper and bilateral lower extremities. On day 13 his left EVD spontaneously occluded and ICP increased to above 30 mmHg (normal range 7 to 15 mmHg). Left EVD was flushed, then replaced and opened at 0 mmHg.

**Fig. 4:**
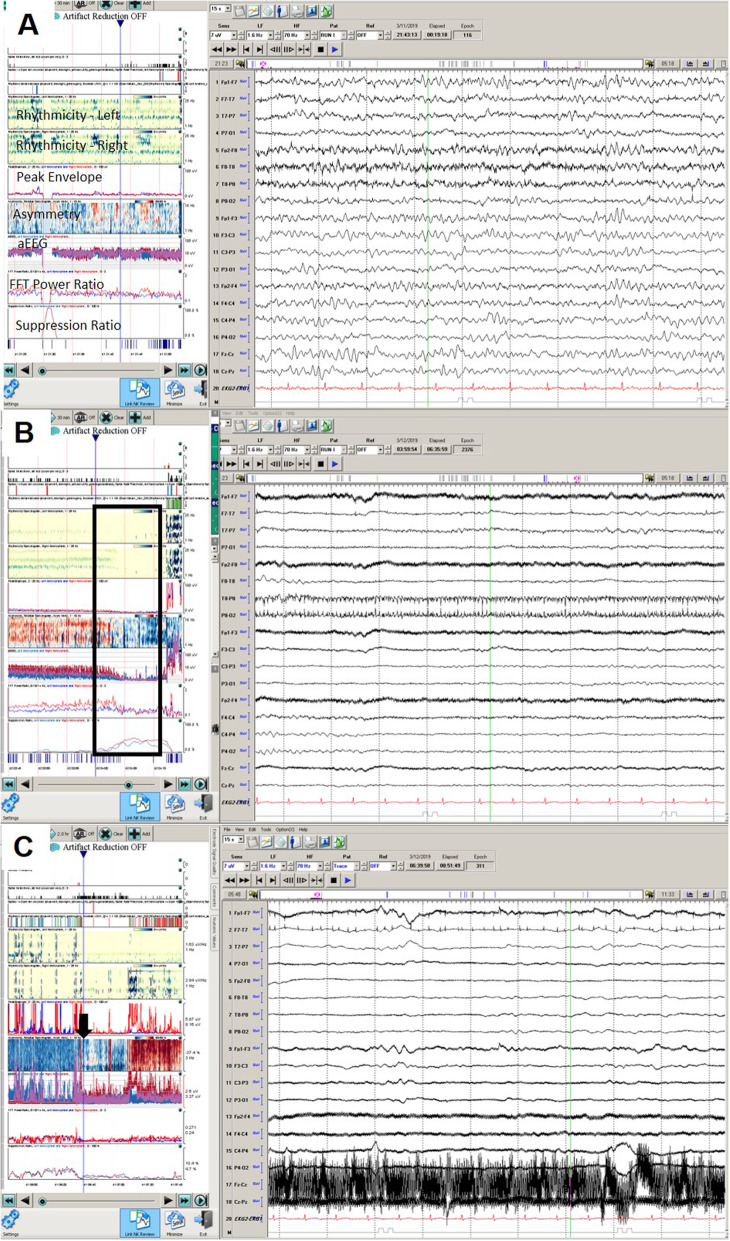
Continuous and quantitative electroencephalography changes of case 2. **a** Baseline EEG showing diffuse slowing, maximum in the left hemisphere. **b** box indicates the area of worsening exam with decrease in rhythmicity especially on the right, worsening asymmetry, and increasing burst suppression ratio. **c** After osmotherapy is administered (black arrow), there is a return in rhythmicity in the right hemisphere and improvement in the asymmetry and reduction in burst suppression ratio

On day 14 at 04:00 am, his exam worsened with fixed and dilated pupils (7 mm; neuroptics). Other brainstem reflexes, including corneal reflex, cough, and gag reflexes were absent. No spontaneous breaths over the ventilator were noted. Twenty minutes prior to changes in clinical examination, QEEG demonstrated loss of rhythmicity, worsening asymmetry, decreasing amplitude, and increasing burst suppression ratio initially in the left hemisphere followed by right hemisphere (Fig. 4); EEG transitioned from continuous generalized/right hemispheric slowing to burst suppression in keeping with brainstem herniation due to acute worsening of hydrocephalus.

He was treated with hyperventilation, hyperosmolar therapy, and replacement of EVDs. At 4:44 am, his pupil size decreased to 4 mm bilaterally but stayed non-reactive. Weak withdrawal in the left upper extremity and triple flexion in bilateral lower extremities were noted. At 5:15 am, right pupil showed reactivity (neuroptics) and his cough reflex returned. At 5:30 am, his cEEG returned to continuous generalized slow pattern. EVD malfunction continued with difficult to control intracranial pressures. Neurosurgery team performed endoscopic ventricular exploration, irrigation, and removal of intraventricular hemorrhage as well as septostomy with placement of new EVD into the third ventricle. Despite aggressive measures, he continued to decline clinically and ultimately expired.

## Discussion and conclusions

In this article, we present two cases supporting the utilization of QEEG as neurotelemetry to detect impending cerebral herniation in the critically ill patient population. In both cases, QEEG showed changes in asymmetry, frequency, amplitude, and finally burst suppression. These changes occurred before clinical recognition.

Using cEEG to monitor patients continuously, particularly those with hemodynamic changes or intracranial hypertension, is established by neurovascular coupling. EEG changes are closely related to cerebral blood flow with faster frequencies (alpha) decreasing and slower frequencies (delta or theta) increasing in ischemia; it has significant direct application to blood pressure augmentation and other time critical interventions [1]. Cerebral perfusion pressure, which is related to intracranial pressure, correlates with surface EEG mean frequency [2, 3]. A case of focal cerebral edema detected on cEEG via hemisphere asymmetry progressing to burst suppression prior to the development of clinical signs has been reported [4]. Chen et al conducted a power spectrum EEG analysis of 62 patents and found a statistically significant negative correlation between the delta power and ICP measured via lumbar puncture (*P* < 0.01) [5]. Indeed, EEG has been successfully used as a non-invasive means of ICP monitoring in a case of coma secondary to cerebral venous sinus thrombosis in which invasive monitoring was contraindicated due to anticoagulation [6], as well as monitoring of cerebral ischemia and herniation [4, 7]. Cyclic patterns can be seen on CEEG in patients with intracranial hypertension. In a recent publication, it was noted that generalized rhythmic delta activity and attenuation of faster frequencies can occur up to 24 h prior to clinical changes [8]. The cyclic nature of these patterns may occur at ~ 1 per minute and ~ 6 per minute corresponding with Lundberg B and C waves, respectively.

The challenge of interpreting large amounts of cEEG data, such as in the concept of neurotelemetry, has been met by computer advances such as Fourier transformation which allow EEG to be quantified in terms of amplitude, frequency, power and rhythmicity [9]. QEEG enables time compressed graphic display arrays to be generated and calculation of power within various frequency bands, percentages of power within a given frequency, and the time during which EEG is composed of given frequencies (i.e. spectral edge). Trending this type of specific data allow various alarm thresholds to be established and use for neurotelemetry, which may allow for earlier intervention and improvement in future outcomes [10–12].

In conclusion, we present two cases highlighting QEEG as neurotelemetry to detect cerebral herniation in the nontraumatic critically ill patient. We document that prior to changes in clinical examination, QEEG demonstrated background slowing, asymmetry alterations, as well as decreases in frequencies and amplitude as much as 2 h prior to onset of burst supression. The use of CEEG and QEEG did not change outcomes in either patient. As the EEG community continues to innovate with the rapid placement of EEG and standardization of interpretation, particularly with machine learning algorithms, we will begin to evaluate the use of CEEG and QEEG on changing outcomes [13]. Using EEG to detect early physiologic harbingers of herniation prior to loss of brainstem function may translate into improved patient outcomes as it may allow earlier intervention. Limitations of this study include its retrospective nature and small sample size limited to non-traumatic patients.

## Data Availability

Not applicable.

## Abbreviations

EEG: Electroencephalography
CT: computerized tomography
ICH: intracerebral hemorrhage
TLS: tumor lysis syndrome
cEEG: continuous electroencephalography
QEEG: Quantitative electroencephalography
IVH: intraventricular hemorrhage
EVD: External ventricular drain
